# Developments in the Management of Metastatic HER2-Positive Breast Cancer: A Review

**DOI:** 10.3390/curroncol29040208

**Published:** 2022-04-08

**Authors:** Julie Lebert, Evan J. Lilly

**Affiliations:** 1 Department of Oncology, Bluewater Health, Sarnia, ON N7T 6S3, Canada; jlebert@bluewaterhealth.ca; 2 Department of Family Medicine, Western University, London, ON N6A 3K7, Canada; 3 Bluewater Health, 89 Norman Street, Sarnia, ON N7T 63S, Canada; 4 Department of Family Medicine, Bluewater Health, Sarnia, ON N7T 6S3, Canada

**Keywords:** breast cancer, metastatic, HER2, trastuzumab, pertuzumab, trastuzumab emtansine, tucatinib, neratinib, trastuzumab deruxtecan

## Abstract

Approximately 20% of breast cancers overexpress human epidermal growth factor receptor 2 (HER2), providing an actionable target for many different therapies. In the metastatic setting, prognosis has improved greatly with the use of anti-HER2 drugs such as trastuzumab, pertuzumab, and trastuzumab-emtansine. In the third line setting and beyond, several emerging treatments have shown benefits, including novel small molecule targeted agents and antibody-drug conjugates. Systemic treatment of brain metastases in HER2-positive patients and the role of endocrine-based treatment for patients with hormone receptor (HR) positive disease remain areas of research interest. This article will review the current approach to systemic management of metastatic HER2-positive breast cancer in Canada, and present novel treatments that may be available in the near future.

## 1. Introduction

Breast cancer remains the most commonly diagnosed cancer in Canadian women, representing 25% of all malignancies diagnosed in women in 2020 [1]. An estimated 15–20% are found to over-express human epidermal growth factor receptor 2 (HER2) [2], and despite advances in treatment, approximately 15–24% will develop metastatic disease after completion of curative-intent treatment, and 3–10% present with de novo metastatic disease [3,4]. The development of trastuzumab, the first HER2-directed monoclonal antibody, greatly improved the prognosis and outcomes of patients with HER2-positive breast cancer, but management remains challenging in the post-trastuzumab era [5]. Additional anti-HER2 treatments, such as pertuzumab and trastuzumab emtansine (T-DM1) have greatly advanced the treatment of this disease. Median overall survival (OS) is now approximately 5 years, and a significant proportion of patients are alive at 8 years [6].

Nonetheless, despite these impressive advances, challenges and questions remain. For example, as more treatment options emerge, optimal sequencing will become an important consideration. Second, central nervous system (CNS) metastases are common, and an important source of morbidity and mortality in patients with breast cancer, but traditional systemic therapy has poor penetration into the CNS. In this rapidly evolving treatment landscape, general practitioners in oncology (GPOs) can play a vital role in helping patients navigate treatment decisions, manage short and long-term adverse effects, and help balance hope for long-term control with pragmatism and preparation. In this review, we discuss current standard and emerging treatments for metastatic HER2-positive breast cancer that could alter and expand the treatment landscape (see Table 1).

## 2. Current Standard Treatments

### 2.1. Trastuzumab, Pertuzumab, and Chemotherapy

The combination of pertuzumab, trastuzumab, and chemotherapy (usually a taxane) is currently considered first-line treatment for most patients with metastatic HER2-positive breast cancer based on the results of the CLEOPATRA trial [6]. This trial randomized patients to receive either pertuzumab or placebo in combination with trastuzumab and docetaxel in the first-line setting. Docetaxel was ideally given for at least 6 cycles, and the other two agents were given until disease progression or toxicity every 3 weeks. The combination of trastuzumab, pertuzumab, and docetaxel led to improved outcomes. Median progression-free survival (PFS) was 18.7 months (95% CI 17–22) in the pertuzumab group, compared to 12.2 months (95% CI 10–14) in the placebo group (HR 0.69, 95% CI 0.59–0.81). Furthermore, the median OS was 57.1 months (95% CI 50–72) in the pertuzumab group compared to 40.8 months (95% CI 36–48) in the placebo group (HR 0·69, 95% CI 0·58–0·82) [7,8,16]. Although the trial was performed in patients without prior treatment for their metastatic disease, benefits were still seen in patients who previously received trastuzumab in the adjuvant setting [7,17]. Notably, patients with CNS metastases were excluded. Alternative chemotherapeutic agents such as paclitaxel or vinorelbine have also been studied in combination with dual anti-HER2 agents [18,19].

In the CLEOPATRA trial, more patients in the pertuzumab group experienced serious adverse effects such as febrile neutropenia, diarrhea, and pneumonia [20]. However, long-term analyses did not reveal any additional risk of cardiac toxicity in the pertuzumab group [7,8,16,20]. Patients should still have their cardiac function monitored in accordance with established recommendations, but practitioners can be reassured that pertuzumab does not amplify cardiovascular morbidity. Practitioners should also be attentive to symptoms of taxane-induced neuropathy during the induction phase of treatment to avoid long-term morbidity, as many patients will live several years after induction.

### 2.2. Trastuzumab-Emtansine

T-DM1 is an antibody-drug conjugate (ADC) that pairs trastuzumab with the cytotoxic microtubule-inhibitor DM1. This structure allows intracellular delivery to HER2-overexpressing cells, thereby enhancing on-target exposure and limiting effects on normal tissue [9]. A stable linker connects both molecules and delivers a “payload” of 3.5 molecules of DM1 per antibody [21]. 

T-DM1 was initially studied in the EMILIA trial, which compared the use of second-line T-DM1 to capecitabine and lapatinib in patients who had previously received trastuzumab and taxane chemotherapy. In the initial trial, median PFS was significantly longer for patients receiving T-DM1 compared to capecitabine and lapatinib (9.6 months vs. 6.4 months; HR 0.65 [95% CI 0.55–0.77]) [9]. Subsequent analyses revealed that median OS was longer with T-DM1 than with control (29.9 months [95% CI 26.3–34.1] vs. 25.9 months [95% CI 22.7–28.3]; HR 0.75 [95% CI 0.64–0.88]) [9,10]. Patients who received T-DM1 also had greater objective response rates, and almost two-thirds of patients were alive after two years [9].

The EMILIA trial also demonstrated T-DM1 to be a comparatively safe treatment. Adverse effects of grade 3 or greater were observed less often in patients treated with T-DM1 than with capecitabine and lapatinib (40.8% vs. 57.0%). The most common adverse effects grade 3 or greater with T-DM1 were thrombocytopenia, followed by elevated aminotransferases. Thrombocytopenia mostly emerged in the first two cycles of treatment, and can usually be managed with dose interruption and/or modification. Fortunately, although rates of bleeding were higher in patients treated with T-DM1, cases of serious bleeding were rare (1–2%). Significant cardiotoxicity was also rare in these patients [9,10].

In Canada, T-DM1 is currently considered the optimal second-line therapy for most patients [22]. However, interim results of the Destiny-Breast03 clinical trial have shown improved ORR and PFS with trastuzumab deruxtecan (T-DXd) in the second-line setting. Encouragingly, responses in patients with brain metastases were significantly higher as well (67.4% vs. 20.5% for T-DM1). Data on OS and mature results are still pending, but T-DXd may soon become the new standard second-line therapy following progression on trastuzumab and pertuzumab-based treatment [12,13]. Data supporting T-DXd in later line treatment is discussed below.

### 2.3. Endocrine Therapy

Approximately 50% of HER2-positive breast cancer patients are also hormone receptor (HR) positive [6]. While HER2-positivity in HR-positive diseases can produce endocrine resistance, HER2 blockade can restore endocrine sensitivity [23,24]. Several studies have evaluated the use of concurrent endocrine therapy and anti-HER2 therapy with no significant safety concerns noted [25,26,27,28]. At this time, the relative benefit of anti-HER2 and endocrine therapy in these patients is uncertain. However, based on current evidence, it seems reasonable to use endocrine therapy in combination with anti-HER2 therapy in this patient population, perhaps even in the first-line setting following induction chemotherapy [6,29].

## 3. Later Line and Emerging Treatments

### 3.1. Small Molecule Tyrosine Kinase Inhibitors

#### 3.1.1. Lapatinib

Lapatinib is a reversible HER1/HER2 tyrosine kinase inhibitor (TKI) that entered the HER2 landscape in 2006 after it was studied in combination with capecitabine in patients previously exposed to anthracycline, taxane, and trastuzumab [14,30]. Lapatinib with capecitabine was compared to capecitabine alone in 399 women and demonstrated improvement in median time to progression (8.4 months with combination therapy vs. 4.4 months with monotherapy; HR 0.57 [95% CI, 0.43–0.77]) and a non-significant trend toward improvement in OS. These improvements were achieved without increased risk of serious toxicity or cardiotoxicity [30]. Follow-up analysis also showed that combination therapy reduced the risk of CNS involvement at the time of progression, and that benefits were seen across all patient subgroups [14]. 

Lapatinib has also been studied as a chemotherapy-free option for patients who have progressed on trastuzumab-containing regimens [31]. This phase III trial studied the efficacy of lapatinib with or without trastuzumab in a heavily pre-treated population; the median number of prior trastuzumab-containing regimens was three. Median PFS was significantly greater in patients receiving lapatinib and trastuzumab compared to lapatinib alone (12.0 weeks vs. 8.1 weeks; HR 0.73 [95% CI 0.57–0.93). Additionally, more than twice as many patients who received combination therapy were progression-free at 6 months (28% vs. 13%) [31].

Common side effects of lapatinib include diarrhea, rash, nausea/vomiting, and fatigue [31]. Diarrhea frequently occurs within the first week of lapatinib initiation and should be managed promptly. Mild cases can be managed with oral hydration, avoidance of irritating foods, and antidiarrheals, as required, while continuing lapatinib. More severe cases may require intravenous hydration and hospital admission, along with temporary or permanent cessation of therapy. The rash seen with lapatinib is the papulopustular (formerly referred to as “acneiform”) rash typical of EGFR inhibitors. It can be managed with emollients, oral tetracycline antibiotics, and avoidance of sun exposure. Notably, significant cardiotoxicity with lapatinib is rare, making it an attractive option in patients receiving other potentially cardiotoxic medications [32].

At the time of writing, lapatinib in combination with capecitabine is most frequently used in Canada in the third-line setting or later since the emergence of T-DM1 and pertuzumab. 

#### 3.1.2. Tucatinib

Tucatinib has a high affinity for HER2 and minimal activity on HER1 [11]. Tucatinib was evaluated in the HER2CLIMB study, in which patients were randomized to receive trastuzumab, capecitabine, and either tucatinib or placebo. All patients had previously received trastuzumab, pertuzumab, and T-DM1 [11]. Almost half of the patients in this trial had brain metastases at enrollment. Compared to placebo, patients who received tucatinib in combination with trastuzumab and capecitabine had a significantly longer PFS (7.8 months [95% CI 7.5–9.6] vs. 5.6 months [95% CI 4.2–7.1]; HR 0.54 [95% CI 0.42–0.71]) and OS (21.9 months [95% CI 18.3–31.0] vs. 17.4 months [95% CI 13.6–19.9]; HR 0.66 [95% CI 0.50–0.88]) [11]. 

Perhaps more importantly in this patient population, the objective response rate (ORR) was significantly higher in patients receiving tucatinib (40.6% overall, 47.3% intracranially) than placebo (22.8% overall, 20.0% intracranially) [11,33]. Furthermore, among patients with brain metastases at baseline, intracranial PFS at 1 year was 25% in patients receiving the tucatinib combination compared with 0% in the placebo combination. These survival benefits were also accompanied by longer preservation of quality of life [11].

The most common adverse effects of tucatinib include diarrhea, palmar-plantar erythrodysesthesia syndrome (PPES), nausea, vomiting, and fatigue. Diarrhea typically occurs within the first two weeks of therapy and should be managed similarly to lapatinib-induced diarrhea. PPES was the most common adverse effect of grade 3 or higher and should be managed in the usual fashion, with dose interruption and/or reduction for more severe cases. Elevations in alanine aminotransferase (ALT), aspartate aminotransferase (AST), and bilirubin were fairly common but mostly low-grade, transient, and reversible upon discontinuation [11]. Higher grade elevations may require dose reduction or cessation. Finally, tucatinib can affect the renal handling of creatinine, which increases serum creatinine without affecting glomerular function. Increases persisted throughout treatment and resolved upon treatment discontinuation [11].

Based on these data, tucatinib, in combination with trastuzumab and capecitabine, may be available for use in Canada for patients with metastatic HER2-positive breast cancer who have received at least one prior line of anti-HER2 therapy, and may be a particularly attractive option for patients with brain metastases [34].

#### 3.1.3. Neratinib

Neratinib is an irreversible inhibitor of HER1, HER2, and HER4 [35]. It has demonstrated efficacy in early-stage HER2-positive breast cancer after adjuvant trastuzumab therapy [36] and effectively prevents and treats brain metastases in HER2-positive metastatic breast cancer [37]. As more treatments for HER2-positive metastatic breast cancer have been discovered, the NALA trial was designed to compare neratinib to lapatinib. In this trial, patients who had received at least two previous lines of treatment were randomized to receive either neratinib or lapatinib (a reversible HER2 inhibitor) plus capecitabine [15]. The study population also included patients with stable and/or asymptomatic CNS disease, but CNS screening was not mandatory. Mean PFS was improved in patients receiving the neratinib combination (8.8 months [95% CI 7.8–9.8]) compared to the lapatinib combination (6.6 months [95% CI 5.9–7.4]), although there was no difference in median PFS. Furthermore, patients who receive the neratinib combination required fewer CNS interventions than patients who received the lapatinib combination (22.8% vs. 29.2%; HR 0.78 [95% 0.60–1.01]). However, there was no significant difference seen in ORR or OS [6,15].

It should be noted that in the NALA trial, only 41.7% of patients had prior exposure to pertuzumab, and 54.3% had prior exposure to T-DM1 [15]. Since these two agents are part of the current first- and second-line standard of care, some uncertainty remains about the efficacy of neratinib after these two agents. Nonetheless, the combination of neratinib and capecitabine may appeal to some patients and providers as there is no infusional component, which allows for more facile delivery. The improved efficacy in delaying or preventing intracranial disease progression is also attractive due to the high morbidity associated with CNS disease. 

The most common adverse effects seen with neratinib include diarrhea, PPES, nausea, and vomiting. Practitioners should be especially attentive to diarrhea because patients receive concomitant capecitabine. To prevent or mitigate significant diarrhea, antidiarrheal prophylaxis with loperamide is required upon initiation [6,15].

Neratinib has recently been approved by Health Canada for use in patients with metastatic HER2-positive breast cancer who have received at least two prior lines of treatment [38].

#### 3.1.4. Pyrotinib

A newer agent, pyrotinib, has been developed as an irreversible pan-HER inhibitor [6], and was investigated in the PHOEBE trial out of China [39]. Patients in this trial had all been previously treated with trastuzumab and taxane chemotherapy, and some had received prior anthracycline chemotherapy. Notably, patients in the PHOEBE trial had not received prior pertuzumab or T-DM1, as these treatments were not approved in China at the time of the trial [39]. Patients were randomized to receive either pyrotinib or lapatinib in combination with capecitabine. In the interim analysis, PFS was found to be significantly improved in the pyrotinib combination compared to the lapatinib combination (12.5 months [95% CI 9.7–NR] vs. 6.8 months [95% CI 5.4–8.1]; HR 0.39 [95% CI 0.27–0.56]). ORR was 67.2% with the pyrotinib combination, compared to 51.5% with the lapatinib combination [39]. However, these results should be interpreted with caution, because patients had not received prior pertuzumab or T-DM1. 

The most common adverse effects seen in the PHOEBE trial were diarrhea and PPES. Diarrhea was significantly more common in patients receiving pyrotinib than lapatinib, whereas rates of PPES were similar between groups [39]. Diarrhea occurs soon after the onset of treatment and dissipated after the first cycle. It can generally be managed with as-needed antidiarrheal treatment, dose interruption, or dose reduction [39]. No suggestions around prophylactic antidiarrheal treatment were made but could be considered in future studies.

At the time of writing, pyrotinib is still pending review by Health Canada, so its place in the Canadian treatment landscape for HER2-positive metastatic breast cancer is uncertain.

### 3.2. Antibodies and Antibody Drug Conjugates

#### 3.2.1. Trastuzumab Deruxtecan (T-DXd)

T-DXd is an ADC with a similar mechanism of action to T-DM1. This particular conjugate consists of an anti-HER2 antibody linked with a topoisomerase I inhibitor. Notably, it has an increased payload compared to T-DM1 (8 vs. 3–4 drug-to-antibody ratio) [40]. Furthermore, its payload easily crosses the cell membrane, which potentially allows for potent cytotoxic effects on nearby cancer cells, the so-called “bystander effect” [41].

T-DXd was evaluated in 184 confirmed HER2-positive metastatic breast cancer patients who were heavily pretreated in the DESTINY-Breast01 clinical trial, a single-arm phase II study [42]. The median of prior treatment lines was 6. In this trial, ORR was 60.9%, median response duration was 14.8 months and median PFS was 16.4 months [42]. While these results were reported after a median follow-up of only 11.1 months, the frequency and duration of response are striking. Based on these encouraging results, T-DXd has received approval for compassionate access in Canada [43]. More recently, T-DXd has been compared head-to-head with T-DM1 in the second-line setting after treatment with the combination of trastuzumab and taxane chemotherapy. Results from the DESTINY-Breast03 study were presented at the European Society for Medical Oncology (ESMO) congress and San Antonio Breast Cancer Symposium in 2021 [12,13]. The data presented show improved response rates (67.4% vs. 20.5% in patients with CNS involvement, and 82.1% vs. 36.6% in patients without CNS involvement), improved PFS (not reached vs. 6.8 months, HR 0.284, *p* < 0.01) and improved OS at 12 months (94.1% vs. 85.9%, HR 0.5546, *p* < 0.01) [13].

Adverse effects were quite common with T-DXd, and in the DESTINY-Breast01 trial, 57.1% of patients had an adverse effect of grade 3 or higher [42]. The most common of these adverse effects were neutropenia (though febrile neutropenia was rare), anemia, nausea, leukopenia/lymphopenia, and fatigue. T-DXd was not associated with cardiotoxicity [42]. A less common but notable adverse effect was pneumonitis, which occurred in 13.6% of patients. Clinicians should educate patients about symptoms of pulmonary toxicity, and promptly respond to patient concerns. Management may involve a combination of drug interruption/cessation, glucocorticoids, supportive care (i.e., supplemental oxygen, symptom management), and Respirology referral [42]. Due to this risk, T-DXd use is contraindicated in patients with a history of interstitial lung disease.

#### 3.2.2. Margetuximab

Margetuximab is a HER2-directed antibody with genetically engineered sequence alteration to optimize antibody-dependent cellular cytotoxicity [44]. Despite this optimization, studies show only modest improvement in PFS. In the SOPHIA phase III trial, margetuximab was compared with trastuzumab, both in combination with chemotherapy, in the third or later line setting [45]. Patients treated with margetuximab had a median PFS of 5.8 months, compared to 4.9 months for patients treated with trastuzumab (HR 0.76 [95% CI 0.59–0.98]). No significant improvement in OS was reported. ORR was higher in patients treated with margetuximab compared to trastuzumab (25% vs. 14%) [45]. A further follow-up analysis is expected in the next year. Though the findings are statistically significant, the clinical significance remains uncertain pending further analysis. At the time of writing, margetuximab has not received Health Canada approval.

The only adverse effect found to be higher among patients receiving margetuximab compared to trastuzumab was infusion-related reactions (IRR). Almost all IRR were isolated to the first cycle of treatment. There was no increased risk of cardiotoxicity compared to trastuzumab [45].

## 4. Exploratory Approaches

In addition to the emerging therapies outlined above, several novel treatment approaches are currently under investigation but not available for use in Canada outside of clinical trials. A novel ADC, trastuzumab duocarmazine (SYD985), is under investigation in a phase III trial comparing SYD985 and trastuzumab, both in combination with chemotherapy. This study is expected to finish in May 2022.

Immune checkpoint inhibition also holds promise as a treatment strategy, because HER2-positive breast cancers frequently have high levels of tumor-infiltrating lymphocytes and programmed cell death protein ligand 1 (PD-L1) expression, similar to other disease sites known to be responsive to immune checkpoint inhibition. Currently, two trials are investigating the use of trastuzumab and T-DM1 in combination with pembrolizumab and atezolizumab, respectively [46,47]. Early results are encouraging, but more work is required to confirm these results, and further define subgroups of patients that respond more or less favorably to these combinations.

The use of cyclin-dependent kinase (CDK) 4/6 inhibitors together with HER2 blockade is also under investigation. In preclinical studies, CDK4/6 inhibitors work synergistically with trastuzumab, and may even resensitize resistant tumors to HER2 blockade [48]. One trial studying patients with triple-positive metastatic breast cancer has reported encouraging results with abemaciclib while another has reported similar results with the use of palbociclib [49,50]. At this stage, the role of CDK4/6 inhibitors in the treatment of patients with triple-positive metastatic breast cancer is uncertain and requires further study. There is also interest in alpha-specific PI3K inhibitors such as alpelisib, as preclinical studies suggest a role for the PI3K pathway in anti-HER2 resistance mechanisms [51]. Several trials are ongoing to explore alpha-specific PI3K inhibition in refractory HER2-positive metastatic breast cancer.

Finally, there is also intense research interest in identifying predictive biomarkers in HER2-positive metastatic breast cancer to enable a more personalized approach to treatment [15,39,45]. This topic is beyond the scope of this review.

## 5. Ongoing Challenges

Despite significant advances made in the treatment of metastatic HER2-positive breast cancer, translating research data into practice presents several challenges. Sequencing of treatment options can become convoluted due to heterogeneity of study populations and accessibility of different agents by jurisdiction. Furthermore, patient-specific factors, such as comorbidities, adverse effect profiles, and patient preferences must also be considered in treatment decisions. As newer agents continue to be developed, more trials will examine the optimal sequencing of agents, and compare the impact of combination treatments to maximize response rates and duration of disease control.

Optimal treatment of patients with both controlled and uncontrolled brain metastases will continue to be a clinical challenge. However, encouraging CNS response rates seen with newer agents provide hope for better disease control, maintenance of the quality of life, and potentially reducing the need for radiation. Progressive disease almost invariably develops during treatment. Resistance pathways and loss of HER2 overexpression can occur. Overcoming resistance with novel targeted agents or multi-agent approaches will be needed. The role of HER2-directed treatment in patients with subsequent loss of HER2 overexpression is also unknown and the best approach for these patients with advanced disease remains unclear.

## 6. The Canadian Treatment Landscape

Currently, the first-line treatment for most patients with metastatic HER2-positive breast cancer in Canada is trastuzumab, pertuzumab, and up to 6 cycles of taxane-based chemotherapy, followed by maintenance trastuzumab and pertuzumab until progression or toxicity. The exception would be patients who have experienced disease recurrence within 6 months of completion of trastuzumab-based adjuvant regimens. For these patients, T-DM1 is likely the best available option at this time.

At the time of progression, the standard second-line treatment has been T-DM1 until progression or toxicity. For patients with CNS metastases, tucatinib plus trastuzumab plus capecitabine would be an excellent alternative due to good CNS penetration and high intracranial response rates. However, T-DXd may soon be approved in the second-line treatment for most patients, including those with CNS metastases, based on data from the DESTINY-Breast03 trial described above. Patients with interstitial lung disease would not be eligible for T-DXd, and would then receive either T-DM1 or tucatinib-based therapy in the second line.

There is considerably less consensus on treatment in the third-line setting and beyond. The authors suggest that patients receive T-DM1 or tucatinib-based therapy in the third-line setting, based on what they received previously. Lapatinib or neratinib plus capecitabine can also be considered in later treatment lines and may be preferable for patients who wish to avoid intravenous treatments. Access could be an important factor in treatment decisions as well; for example, lapatinib plus capecitabine is generally government-funded, whereas neratinib plus capecitabine is not, and would require third-party coverage or compassionate access to be feasible for most patients.

Once anti-HER2 treatments and small molecule TKIs are exhausted, patients can be considered for traditional cytotoxic chemotherapy. However, these treatments are limited by poor response rates and high toxicity. For fit and interested patients, clinical trials should also be considered at this stage.

**Figure 1:**
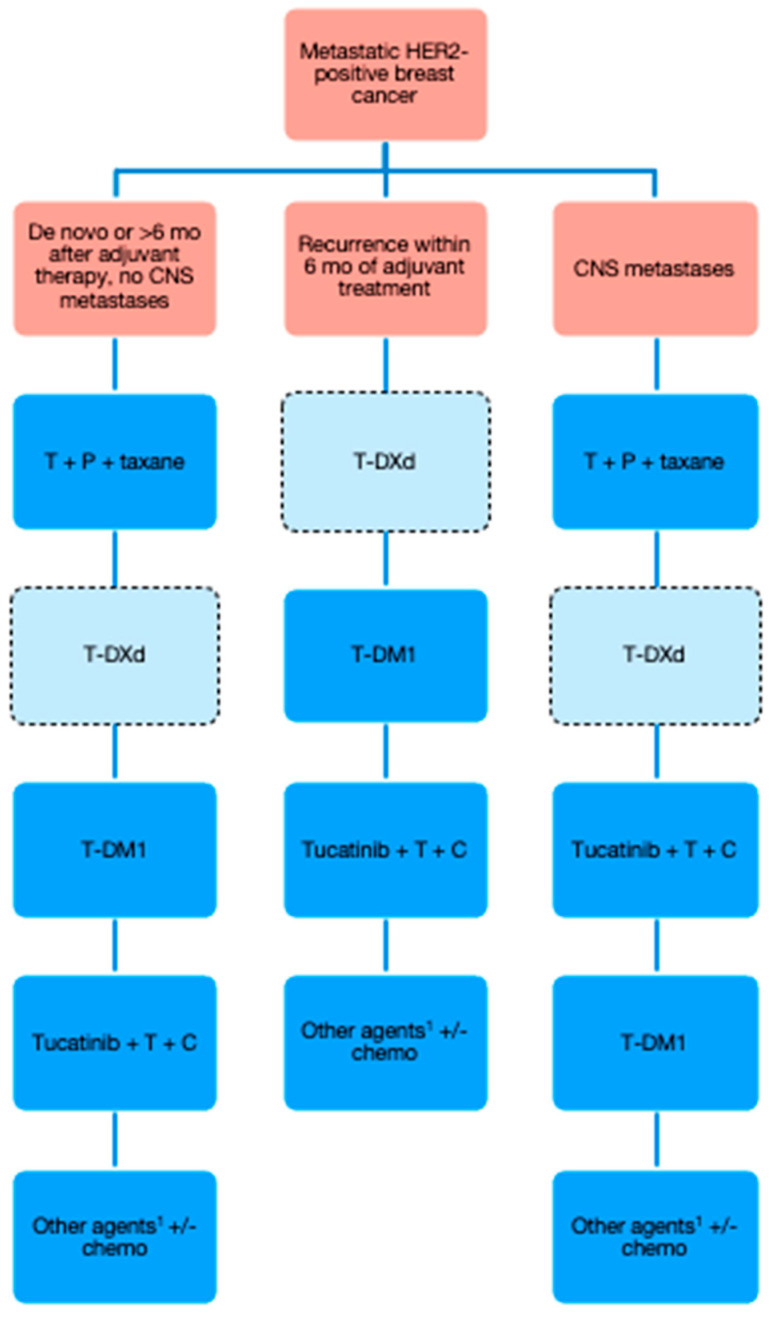
Proposed algorithm for publicly funded treatment of HER2-positive metastatic breast cancer in Canada. CNS = central nervous system; T = trastuzumab; P = pertuzumab; T-DXd = trastuzumab deruxtecan; T-DM1 = trastuzumab emtansine; C = capecitabine. ^1^ Clinical trials should always be considered at this stage as well.

A proposed algorithm for the sequencing of publicly funded treatment options in Canada is presented in Figure 1. T-DXd is entered in the lighter-colored box with hashed borders to indicate its pending approval status for second-line treatment.

## 7. Conclusions

In recent years, several new treatments have emerged for patients with HER2-positive metastatic breast cancer, and outcomes have consequently improved. Nonetheless, despite exciting developments in the treatment of metastatic HER2-positive breast cancer in the last several years, this disease continues to be the cause of mortality in most patients with rare chances for cure or long-term survival. Future research will investigate the use of immune checkpoint inhibitors, endocrine blockade, CDK4/6 inhibitors, and PI3K inhibitors alongside methods of HER2 blockade. Research into predictive biomarkers may identify subgroups of patients who respond differently to various treatments, allowing personalization of their individual treatment algorithms. These advances and research frontiers offer clinicians optimism for a better future for their patients with HER2-positive metastatic breast cancer.

## Figures and Tables

**Table 1:** Health Canada-approved novel therapies for HER2-positive metastatic breast cancer.

Drug	Route	Frequency	Combination	OS Benefit
Pertuzumab	IV	q3wk	Trastuzumab + taxane	15.7 months [7,8]
T-DM1	IV	q3wk	None	6.8 months [9,10]
Tucatinib	PO	Daily	Trastuzumab + capecitabine	4.5 months [11]
T-DXd	IV	q3wk	None	Likely, but awaiting further analysis [12,13]
Lapatinib	PO	Daily	Capecitabine	None [14]
Neratinib	PO	Daily	Capecitabine	None [15]
